# Harmful Effects of Pyraclostrobin on the Fat Body and Pericardial Cells of Foragers of Africanized Honey Bee

**DOI:** 10.3390/toxics10090530

**Published:** 2022-09-09

**Authors:** Lais V. B. Inoue, Caio E. C. Domingues, Aleš Gregorc, Elaine C. M. Silva-Zacarin, Osmar Malaspina

**Affiliations:** 1 Centro de Estudos de Insetos Sociais (CEIS), Departamento de Biologia Geral e Aplicada, Instituto de Biociências (IB), Universidade Estadual Paulista (UNESP)-“Júlio de Mesquita Filho”, Rio Claro 13506-900, SP, Brazil; 2 Faculty of Agriculture and Life Sciences, University of Maribor, Pivola 10, 2311 Hoče, Slovenia; 3 Laboratório de Ecotoxicologia e Análise de Integridade Ambiental (LEIA), Departamento de Biologia (DBio), Universidade Federal de São Carlos (UFSCar), Sorocaba 18052-780, SP, Brazil

**Keywords:** *Apis mellifera* L., biomarkers, morphophysiology, residual concentrations, strobilurin fungicide

## Abstract

Managed honey bees are daily exposed in agricultural settings or wild environments to multiple stressors. Currently, fungicide residues are increasingly present in bees’ pollen and nectar and can harm colonies’ production and survival. Therefore, our study aimed to evaluate the effects of the fungicide pyraclostrobin on the fat body and pericardial cells of Africanized honey bees. The foragers were divided into three experimental treatment groups and two controls: pyraclostrobin 0.125 ng/µL (FG1), 0.025 ng/µL (FG2), 0.005 ng/µL (FG3), untreated control (CTL), and acetone control (CAC). After five days of oral exposure (*ad libitum*), the bees were dissected and prepared for histopathological and morphometric analysis. The FG1-treated bees showed extensive cytoarchitecture changes in the fat body and pericardial cells, inducing cell death. Bees from the FG2 group showed disarranged oenocytes, peripheral vacuolization, and pyknotic nuclei of pericardial cells, but the cytoarchitecture was not compromised as observed in FG1. Additionally, immune system cells were observed through the fat body in the FG1 group. Bees exposed to FG3 demonstrated only oenocytes vacuolization. A significant decrease in the oenocyte’s surface area for bees exposed to all pyraclostrobin concentrations was observed compared to the CTL and CAC groups. The bees from the FG1 and FG2 treatment groups presented a reduced surface area of pericardial cells compared to the controls and the FG3 group. This study highlighted the harmful effects of fungicide pyraclostrobin concentrations at the individual bee cellular level, potentially harming the colony level on continuous exposure.

## 1. Introduction

The honey bee (*Apis mellifera* Linnaeus, 1758) is a managed species worldwide. It is recognized for its economic importance due to its pollination in agricultural landscapes used for human consumption and its high honey production [1,2]. Furthermore, due to the generalist profile and adaptation to different environments, it is also relevant in the maintenance of various natural ecosystems [3,4], as well as native bee species [5] and wild pollinators [6]. Nevertheless, the pollination services provided by bees may be threatened due to exposure to multiple stressors and interactions between them, such as deforestation (habitat loss), monocultures, diseases, pesticides, and others [7]. From this point of view, the fact mentioned above is worrying since numerous studies report severe losses in honey bee colonies and wild bees on different continents [8,9,10,11,12,13].

This reality becomes even more concerning as records of the diversity and richness of bees on a global scale suggest that they have been decreasing sharply since the 1990s [14]. Related to this, according to the review by Sharma et al. [15], pesticide use has grown worldwide, and the trend is that users will continue to increase in the coming years, with herbicides, insecticides, and fungicides being the most used classes. In that regard, bees can be exposed during foraging or ingest resources necessary for their development containing pesticide residues, compromising their fitness [16,17].

One of the world’s ten most significant pesticide users is Brazil [15], with significant dependence on these chemicals in the most varied crops. According to Pires et al. [18], the cases of weakening of colony loss in Brazil may be related to the current model of agriculture in the country, loss of forest habitats, and, obviously, the use of pesticides. Due to the flexibility in pesticide regulation and legislation, there are many knowledge gaps regarding the risk of exposure to bees, which does not provide enough protection to them [19]. Among pesticides, insecticides are the most studied class in bees [20]. Cullen et al. [21] highlighted the growing interest in fungicides studies, but many knowledge gaps still need to be elucidated. Corroborating, the review conducted by Rondeau and Raine [22] showed that the distribution of studies with fungicides is more developed in North America and Europe, and there is a great need for more data in other regions.

Although fungicides cause less lethal effects than insecticides and are therefore considered less harmful to nontarget organisms, studies have highlighted their sublethal effects in larvae and adults of bees [23,24,25,26,27,28]. The fungicide pyraclostrobin is widely used in Brazil on more than 130 crops through foliar or seed application, according to the Brazilian Health Regulatory Agency (ANVISA in Portuguese) [29]. Pyraclostrobin belongs to the family of strobilurins that act by inhibiting the mitochondrial respiration of fungus [30]. Some studies have shown adverse effects of pyraclostrobin on honey bees, such as mitochondrial respiratory inhibition [26], reduction of honey bee forager’s longevity [31], cytotoxic effects in the midgut [24,31,32], decreased polysaccharides and midgut proteins [31], higher intensity of chitin marking [24] and compromising the post-embryonic development [23,24].

According to Zioga et al. [33], the fungicide pyraclostrobin is often found in the pollen and nectar of cultivated and wild plant species (arable field margins) visited by honey bees. Knowing the risk of exposure to oral feeding, it is essential to define target organs that participate in the route of absorption and metabolism for use in ecotoxicological studies [34]. It is supposed that the fat body (trophocytes and oenocytes) and pericardial cells are important as target cells in bees for analyzing sublethal effects, since they participate in the central intermediary metabolism in insects such as multifunctional tissue and have pinocytic and phagocytic activity (excretory function), respectively [35,36]. Additionally, according to Abdalla and Domingues [37], the fat body and pericardial cells associated with the immune system are part of an integrated system highly responsive to chemical stressors called the Hepato-Nephrocitic System (HNS). The authors described that the fat body acts as the first barrier against different xenobiotics, followed by the pericardial cells (second barrier) and the immune system cell activity throughout the process.

For this purpose, due to a lack of knowledge about the sublethal effects of fungicides and due to the high importance of the detoxification pathway of the biomarker selected in the present work, our study aimed to evaluate the effects of feeding concentrations of pyraclostrobin on the morphophysiology of the fat body and pericardial cells of Africanized honey bee foragers through an integrated methodology.

## 2. Materials and Methods

### 2.1. Honey Bee Collection

**Figure 1:**
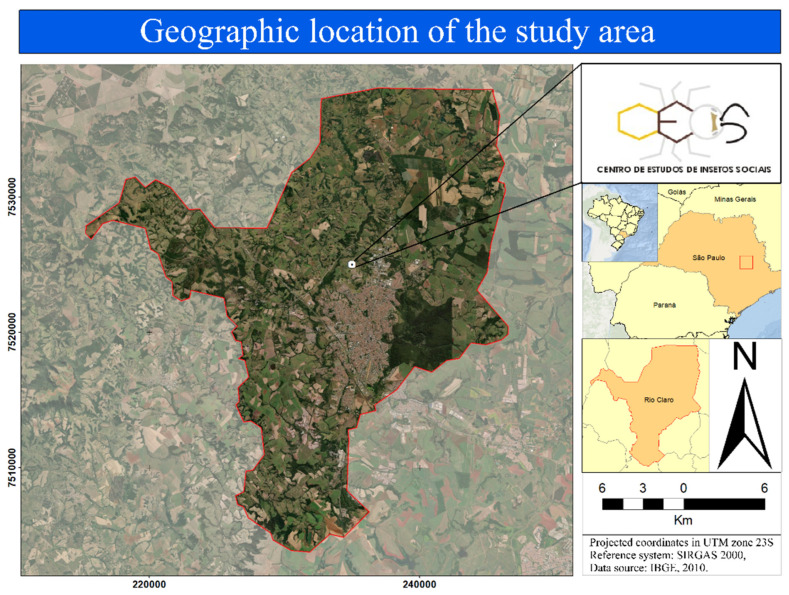
Location of the “Centro de Estudos de Insetos Sociais (CEIS)” in the Rio Claro municipality, where ecotoxicological studies were carried out with foragers of Africanized honey bee.

The present research used six colonies of Africanized honey bees (Langstroth hives) with standardized strength and visually healthy, free of symptomatic diseases and pests. All these colonies were installed in the apiary of São Paulo State University “Júlio de Mesquita Filho” (22°23′48.1″ S; 47°32′33.1″ W), which is located in the municipality of Rio Claro, in the countryside of the state of São Paulo, Brazil. These colonies were kept in an urban area in order to avoid the bees collected being exposed to pesticides by the local farmers. The ecotoxicological bioassay was performed at the Laboratório de Ecotoxicologia e Conservação de Abelhas (LECA), located at the Centro de Estudos de Insetos Sociais (CEIS), from the same aforementioned institution (Figure 1).

Foraging bee collection consisted of inserting bee cages of 250 mL (plastic pots, 9 cm × 7 cm) at the entrance of three different colonies at the selected foraging time (7:00–9:00 a.m.) and with temperatures from 20 °C during the summer season of 2018 in Brazil. These cages were previously prepared with small holes using a needle (1.20 × 40 mm) around them for air circulation, such as filter paper at the bottom of the plastic pots for better hygiene of the place, a larger hole in the lid for the removal of dead bees and a microtube in the lid, with four small holes (0.70 × 30 mm), for the food introduction. Immediately after foraging bee collection, polypropylene microtubes (Eppendorf™, Hamburg, Germany, 2 mL) were filled with syrup (50% water and 50% sugar, *w*/*w*) and added to all bee cages at the LECA.

### 2.2. Chemical

The fungicide pyraclostrobin Pestanal^®^ (CAS Number 175013-18-0, ≥98.0% purity, analytical standard) was purchased from Sigma-Aldrich (Saint Louis, MO, USA). A stock solution (1000 ng a.i./mL) was prepared using acetone (100% purity) and autoclaved distilled water in the proportion of 40–60%, respectively. After that, serial dilutions were performed to obtain working concentrations for the oral exposure to Africanized honey bees. These are based on previous research conducted in our group with *A. mellifera* [31] and *Melipona scutellaris* (Latreille, 1811) [38], as well as the residues range found in nectar and pollen [39,40]. 

### 2.3. Experimental Design-Toxicological Bioassays

After the foraging honey bee collection (described in Section 2.1), the bees were placed in an incubator with a temperature of 33 °C (± 1), a humidity of 70% (±5), and under dark conditions. Four to six hours before oral exposure, the microtubes containing syrup were removed, and dead or reduced-mobility bees were carefully replaced. Thus, the bees were divided—20 bees/cage and five replicates were tested/treated, totaling 100 bees/treatment—into the following experimental groups: pyraclostrobin 0.125 ng/µL-125 ppb in syrup (FG1), pyraclostrobin 0.025 ng/µL-25 ppb in syrup (FG2), pyraclostrobin 0.005 ng/µL-5 ppb in syrup (FG3), control (CTL) and acetone control (CAC). Bees in control groups (CTL and CAC) received syrup without adding fungicide. The CAC was included due to the low solubility of the fungicide pyraclostrobin in water (1.9 mg/L at 20 °C) and the need to use an organic solvent. The final acetone concentration did not exceed 1% of the final volume as described in Organization for Economic Co-operation and Development (OECD) guideline 213 [41]. This work defined oral exposure time (*ad libitum*) as five days, based on Domingues et al. [31]. The bees were dissected and prepared for histological and morphometric analysis after exposure.

### 2.4. Histological Processing of the Fat Body

For the histological procedure, five foragers bees were randomly collected from each experimental group and anesthetized at a cold temperature (4 °C) for one minute. Then, the bees’ parietal fat body was dissected with a digital stereo microscope (LEICA EZ4 HD) and immersed in a fixative solution (paraformaldehyde 4% in phosphate-buffered saline (PBS), 0.1 mol L^1^, pH 7.4) for 24 h at 4 °C. Following the fixation period, these organs were immersed in PBS (pH 7.2–7.6 at 25 °C) for one hour and gradually dehydrated in ethanol, according to Silva-Zacarin et al. [42]. Upon these procedures, all organs were embedded in historesin (Historesin Embedding Kit, Leica Biosystems Nussloch GmbH, Heidelberger Str. 17-19) according to Leica Biosystems’ instructions. After complete polymerization of historesin, organs from five individuals from each group were fixed in wooden cubes (1 cm × 1 cm × 1 cm) and, using a microtome (LEICA RM2255), histological sections of 6 µm were performed gradually in the longitudinal direction of the organ. Histological sections were placed on microscopies slides, stained using the hematoxylin and eosin (HE) technique [43], and fixed to coverslips using dibutylphthalate polystyrene xylene (DPX) mounting medium for microscopic examination (Sigma-Aldrich, Saint Louis, MO, USA, 06522).

### 2.5. Qualitative Analysis of Fat Body and Pericardial Cells 

The analysis and photo documentation of slides was performed using bright-field light microscopy (Olympus BX51) by the program DP Manager Software (Olympus). In order to define the morphological pattern of each experimental group and guarantee a high-accuracy histopathological diagnosis, six slides each with 12 nonsequential histological sections at different depths were made for each individual (n = 5 per group), which resulted in 72 analyses per individual and 360 for each experimental group [25]. On average, 125 images/group were documented, and it was possible to observe the dorsal vessel, lumen, pericardial cells, trophocytes, oenocytes, and immune system cells.

### 2.6. Morphometry of Oenocytes and Pericardial Cells 

The morphometry of parietal fat body oenocytes and pericardial cells was performed by analyzing histological sections in slides stained using the HE technique (described in Section 2.4). Taking advantage of the abovementioned photos (described in Section 2.5), 40x objective (Olympus BX51), 20 measurements for pericardial cells, and 10 for oenocytes of the best-preserved organ sections for each slide (n = 25 per experimental group) were selected along the dorsal vessel. Five individuals were utilized per experimental group. The area of each image was standardized at 35,995.2 µm^2^. In the end, a total of 2.500 pericardial cells and 1.250 oenocytes were evaluated in each experimental group through the ImageJ software bundled with 64-bit Java 1.8.0_172. From this point of view, were measured 12.5 times more pericardial cells and 6.25 times more oenocytes than suggested by Balsamo et al. [44], on which this analysis was based and adapted.

### 2.7. Statistical Analysis

The data from morphometric analyses were previously verified for normal distribution by the D’Agostino–Pearson test. After the previous analysis, the data did not show the normal distribution and, for this reason, were analyzed by the Kruskal–Wallis nonparametric test, followed by Dunn’s multiple comparisons test using the software GraphPad Prism 9.4.0 (673) (GraphPad Prism Software, Inc., San Diego, CA, USA). The graphs presented the results as mean ± standard error, and the significance level adopted was *p* < 0.05.

## 3. Results

### 3.1. Morphological Analyses of Fat Body Cells

**Figure 2:**
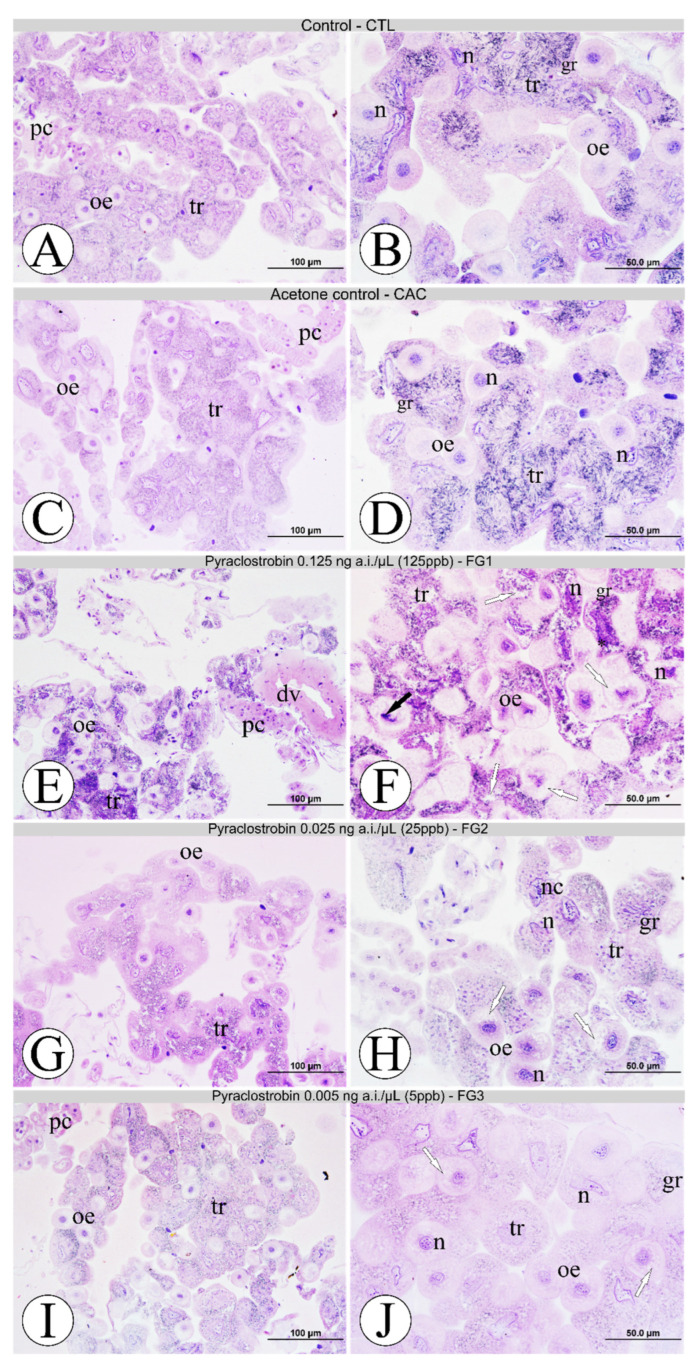
Fat body of foragers of Africanized honey bee after five days of oral exposure to fungicide pyraclostrobin. (**A**,**B**): control-CTL; (**C**,**D**): acetone control-CAC; (**E**,**F**): pyraclostrobin 0.125 ng/µL (125 ppb)-FG1; (**G**,**H**): pyraclostrobin 0.025 ng/µL (25 ppb)-FG2; (**I**,**J**): pyraclostrobin 0.005 ng/µL (5 ppb)-FG3. **Legend**: asterisk = heterochromatin on the periphery, black arrow = nuclei with condensed chromatin, dv = dorsal vessel, gr = granules, n = nuclei, nc = nucleolus, oe = oenocytes, pc = pericardial cells, tr = trophocytes, white arrow = vacuolizations. Histological sections stained with hematoxylin and eosin. N = 5 individuals per experimental group.

The morphological pattern of fat body oenocytes and trophocytes from all experimental groups is summarized in Figure 2. The bees from the CTL and CAC groups showed similar cytoarchitecture and distribution of the fat body cells around the dorsal vessel. Oenocytes from these groups showed standard morphology, with spheroidal cells, centralized nuclei with the presence of several nucleoli, decondensed chromatin, well-defined cell boundaries, and were generally distributed among the trophocytes or associated with them (Figure 2A–D). Trophocytes showed their characteristic irregular morphology with undefined cell boundaries, branched nuclei with nucleoli and condensed chromatin on the edge of the nuclear envelope, acidic and basic granules in the cytoplasm, and the presence of small vacuoles (Figure 2A–D). Bees in the FG1 treatment group expressed a collapsed fat body from extensive cell morphological changes compared to the CTL, CAC, and FG3 groups (Figure 2E,F). The oenocytes lost their standard spherical cytoarchitecture, showed many vacuolizations in the cytoplasm, and decentralized nuclei with condensed chromatin intensive staining with hematoxylin were observed (Figure 2F). The trophocytes showed their cytoarchitecture as disrupted, the nuclei losing the branched feature, the intense presence of acidic granules in the cytoplasm (vacuolated) strongly labeled for hematoxylin, and clumps of heterochromatin on the periphery (Figure 2F). In the FG2 group, oenocytes showed lost spheroidal arrangement, several vacuoles on their cytoplasm, and altered nuclei morphology (Figure 2H). As for trophocytes, they showed fewer injured examples, but it was possible to observe intensely stained nucleoli in the nucleus less branched than its morphological pattern (Figure 2G,H). Bees from the FG3 group showed only a few sporadic oenocytes vacuolization, but the fat body was mainly morphologically more similar to controls than other fungicide pyraclostrobin concentrations (Figure 2I,J).

### 3.2. Morphometric Analyses of Oenocytes

**Figure 3:**
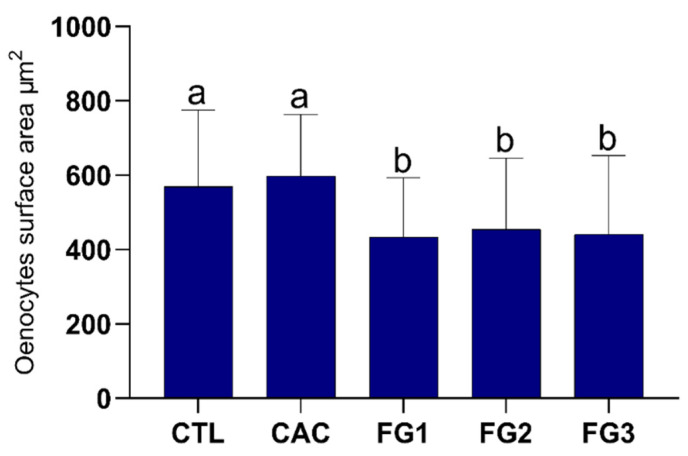
Graph of the surface area of oenocytes from foragers of Africanized honey bee after five days of oral exposure to different treatments. Letters represent significant differences between the experimental groups by the Kruskal–Wallis nonparametric test, followed by Dunn’s multiple comparisons test (*p* < 0.05). Kruskal–Wallis statistic = 125.7. N = 5 individuals per experimental group.

The morphometric results showed a highly significant decrease in the oenocytes surface area for bees exposed to all pyraclostrobin concentrations compared to the CTL and CAC groups (*p* < 0.0001, Kruskal–Wallis statistic = 125.7), as shown in Figure 3. There were no differences (*p* > 0.9999) in the oenocytes surface area between the groups exposed to pyraclostrobin concentrations (FG1, FG2, and FG3). Bees from the control groups had similar oenocytes surface areas (*p* = 0.3750).

### 3.3. Morphological Analyses of Pericardial Cells

**Figure 4:**
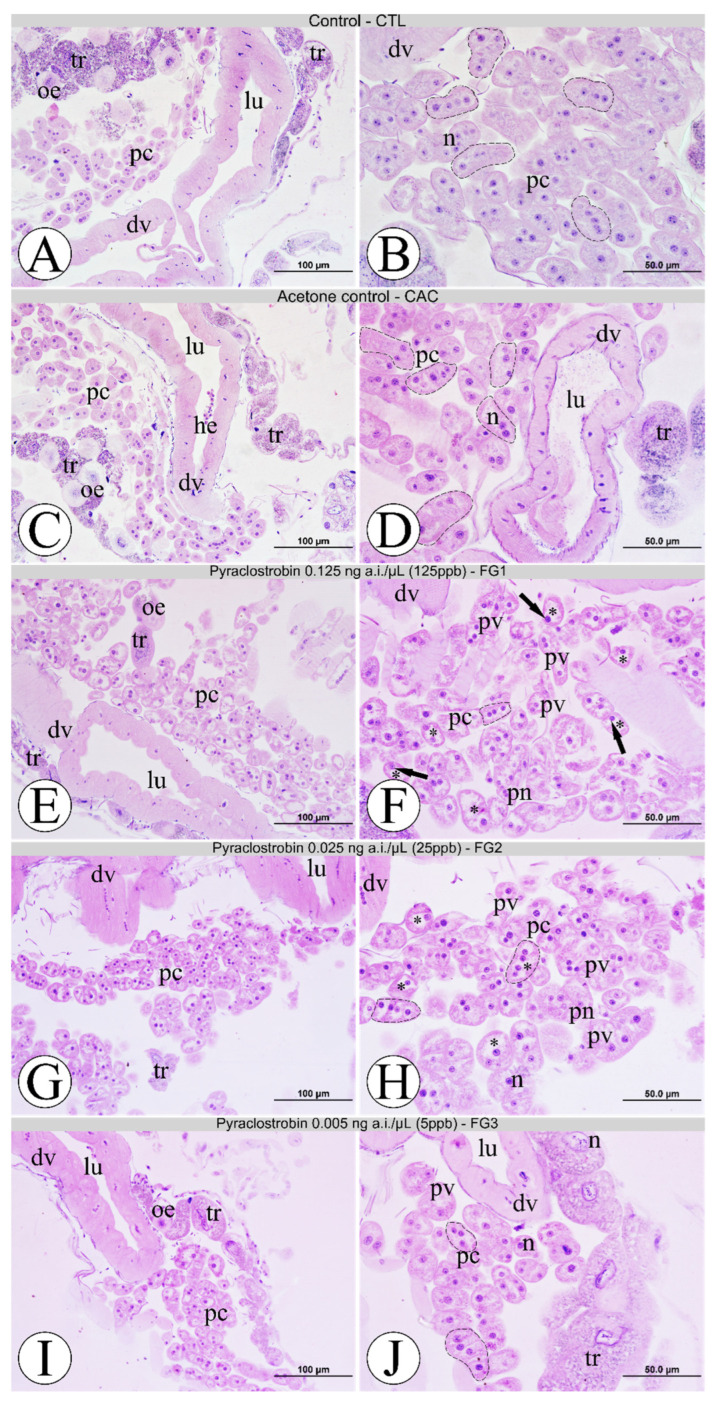
Pericardial cells of foragers of Africanized honey bee after five days of oral exposure to fungicide pyraclostrobin. (**A**,**B**): control-CTL; (**C**,**D**): acetone control-CAC; (**E**,**F**): pyraclostrobin 0.125 ng/µL (125 ppb)-FG1; (**G**,**H**): pyraclostrobin 0.025 ng/µL (25 ppb)-FG2; (**I**,**J**): pyraclostrobin 0.005 ng/µL (5 ppb)-FG3. **Legend**: asterisk = large central vacuole, black arrow = peripheral nucleus, dotted line = cordonal pericardial cells, dv = dorsal vessel, he = hemocytes, lu = lumen, n = nuclei, oe = oenocytes, pc = pericardial cells, pn = pyknotic nucleus, pv = peripheral vacuoles, tr = trophocytes. Histological sections stained with hematoxylin and eosin. N = 5 individuals per experimental group.

Based on the histological analysis of the pericardial cells in the dorsal vessel region of the forages, after the period of exposure to the fungicide pyraclostrobin, it was possible to determine the histomorphology pattern of all experimental groups (Figure 4). Individuals from the CTL and CAC groups did not show morphological changes among themselves, showing pericardial cells with typical morphology, a centralized rounded nucleus, few or the absence of peripheral vacuoles, and cordonal arrangement of stage I (two up to six cells) along the dorsal vessel region, in which it demonstrated pattern histoarchitecture of the muscular wall (Figure 4A–D). Bees from the FG1 group showed extensive morphological changes in pericardial cells with peripheral and mainly central vacuoles displacing the nucleus to the periphery; the cordonal arrangements (stages III and IV) showed irregular and uncharacterized morphology when compared with the CTL and CAC groups (Figure 4E,F). The nuclei strongly labeled for hematoxylin and pyknotic were also observed (Figure 4F). In the FG2 group, the pericardial cells were vacuolized with pyknotic nuclei (stages II and III). However, the cytoarchitecture of these cells was not highly compromised, as observed in bees from the FG1 group (Figure 4G,H). Regarding the FG3 group, the bees showed similar histomorphology patterns in pericardial cells (stage I) and the dorsal vessel compared to the CTL and CAC groups (Figure 4I,J).

### 3.4. Morphometric Analyses of Pericardial Cells

**Figure 5:**
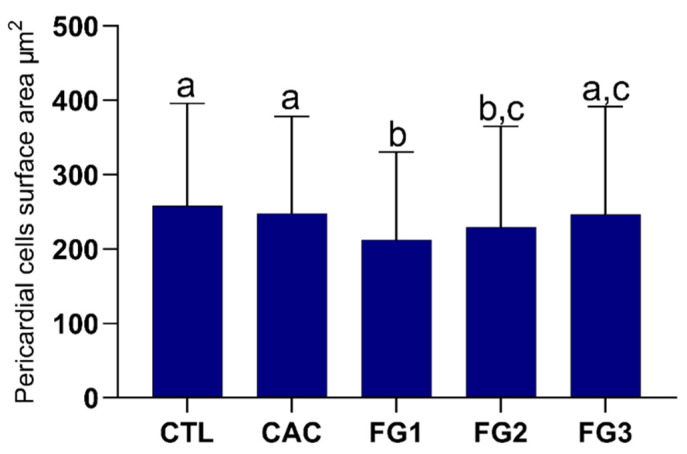
Graph of the surface area of pericardial cells from foragers of Africanized honey bee after five days of oral exposure to different treatments. Letters represent significant differences between the experimental groups by the Kruskal–Wallis nonparametric test, followed by Dunn’s multiple comparisons test (*p* < 0.05). Kruskal–Wallis statistic = 43.33. N = 5 individuals per experimental group.

The bees from the FG1 (*p* < 0.0001) and FG2 (*p* < 0.001) groups presented a reduced surface area of pericardial cells (Kruskal–Wallis statistic = 43.33) compared to the controls, being that FG1 showed a smaller size among all experimental groups (Figure 5). There was no difference in the pericardial cells surface area between FG2 and FG3 (*p* = 0.3262), as well as FG3 and the control groups (CTL vs. FG3, *p* = 0.3161 and CAC vs. FG3, *p* > 0.9999). The control groups had similar pericardial cells surface areas (*p* > 0.9999).

### 3.5. Immune System Cells

**Figure 6:**
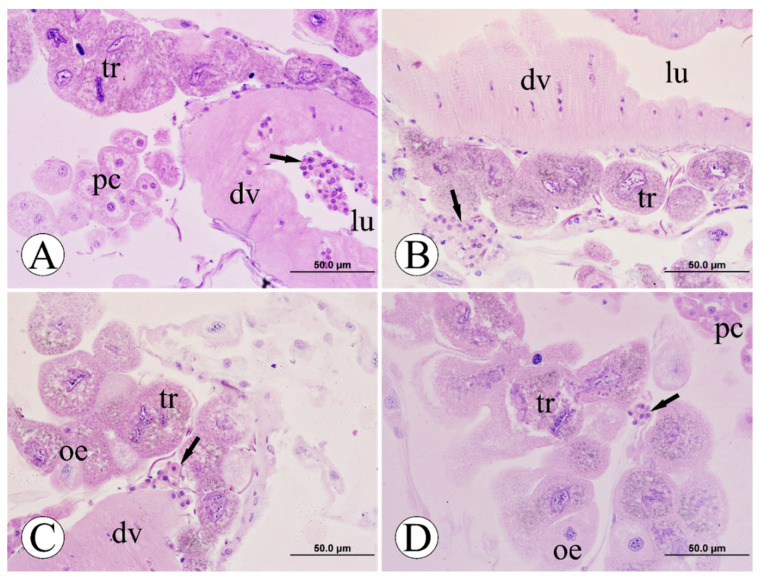
Hemocytes of foragers of Africanized honey bee after five days of oral exposure to fungicide pyraclostrobin. (**A**–**D**): pyraclostrobin 0.125 ng/µL (125 ppb)-FG1. **Legend:** black arrow = agglomeration of immune system cells-hemocytes, dv = dorsal vessel, lu = lumen, oe = oenocytes, pc = pericardial cells, tr = trophocytes. Histological sections stained with hematoxylin and eosin. N = 5 individuals.

In addition to the morphological analyses performed and mentioned above, it was observed only in bees of the FG1 group—highest pyraclostrobin concentration—a cluster of cells of the immune system (hemocytes) located in the lumen of the dorsal vessel (Figure 6A), between the fat body trophocytes and oenocytes (Figure 6B,D) and also surrounding the dorsal vessel region (Figure 6C). No other pyraclostrobin-treated bees showed clusters of immune system cells like in the FG1 group; only a few isolated cells were observed.

## 4. Discussion

Our study revealed that oral exposure to the fungicide pyraclostrobin, even at residual concentrations, causes histopathological and morphometric injuries to the fat body and the pericardial cells of foragers of Africanized honey bees. These findings are relevant as they contribute to reducing the knowledge gap on the effects of fungicides on bees in undeveloped regions, such as South America (Brazil), with extensive beekeeping practice on one side and high use of pesticides in agriculture on the other side [18], along the same lines as the critical research gaps pointed out by Rondeau and Raine [22]. 

Considering that the effects observed here in forager bees were obtained in a laboratory with exposure-controlled conditions, maintaining the colony balance tends to be threatened in a realistic field scenario. According to Domingues et al. [31], foragers of Africanized honey bee exposed continuously to the pyraclostrobin had an 18.75% reduction in survival time compared to the control group. In contrast, the newly emerged bees did not have longevity affected, demonstrating greater sensitivity of older bees to the fungicide. The foragers are responsible for identifying foraging sites, transmitting this information to other workers by waggle dance, and collecting the resources necessary for the colony’s development [45]. Thereby, a decay in foragers’ performance can generate stress in the colony, which is a realistic scenario that can make it more vulnerable to other factors, unbalancing and threatening its maintenance [46].

In accordance with recent literature, other sublethal studies have also shown adverse effects of pyraclostrobin on different bees and organs such as the midgut, mandibular and hypopharyngeal glands of Africanized honey bee [31,47], midgut of Brazilian native stingless bee *Melipona scutellaris* (Latreille 1811) [38] and on the fat body of the neotropical solitary bee *Tetrapedia diversipes* (Klug 1810) [22]. Our data corroborate with Abdalla and Domingues [37], highlighting the sensitivity and efficacy of fat body and pericardial cells as biomarkers for environmental stress caused by pesticides.

Regarding the fat body, this tissue can be located below the integument (parietal) or between the organs (visceral) and is mainly composed of two cell types: trophocytes of mesodermal origin and oenocytes of ectodermal origin [48,49]. It is considered a multifunctional organ in which the production and storage of reserves (carbohydrates, lipids, and proteins) can be highlighted [35,48,49], acting in the composition of hemolymph [50] and metabolism of xenobiotics [19,37,44]. On the other hand, pericardial cells are distributed along the dorsal vessel and related to excretory activity [37,48]. According to Abdalla and Domingues [37], the HNS formed by the interaction between the fat body and pericardial cells with the immune system cells response and a temporal cascade of events. From this point of view, our data for the highest concentration of pyraclostrobin followed the same logic proposed by the authors, where oenocytes and trophocytes were more sensitive to the fungicide (first barrier), followed by pericardial cells (second barrier) and associated hemocytes through this process.

The morphological changes observed in oenocytes and trophocytes in bees exposed to the highest concentration of pyraclostrobin suggest a process of cell death due to the stress involved by the fungicide, since it noticed strongly condensed chromatin, a fragmented nucleus, intense vacuolization, and cytoarchitecture disarrangement. However, these effects were not observed by Domingues et al. [51] after oral exposure to picoxystrobin (strobilurin fungicide) in newly emerged workers of Africanized honey bee at 0.018 ng a.i./µL (18 ppb). It seems that the effects observed here are related to the age of the bees (older bees) and the concentration 7x higher than that used by Domingues et al. [51], but within the range of residues found in pollen and nectar [39,40].

Oenocytes are responsible for bee detoxification (enzymes) and homeostasis [48]. According to Cousin et al. [52], these have high chemical sensitivity, which can be directly correlated to the statistically significant difference in the surface area of oenocytes in bees exposed to the fungicide. From this perspective, the same effects were also observed in *A. mellifera* after exposure to the insecticide thiamethoxam, *Nosema ceranae* (microsporidian) inoculation [44], and the herbicide paraquat [52]. The trophocytes are essential for providing energy for the metabolism and synthesis of hemolymph proteins [35,48,53], which is essential for the development and survival of individuals. However, exposure to fungicide can cause damage to cells since it manifests morphological changes in the regularity of membranes and nuclei compared to the control groups. In addition, these data corroborate studies carried out by Assis et al. [19], which demonstrate that pyraclostrobin increases the number of vacuoles in cells and influences an atypical morphology of trophocytes.

In addition to the earlier changes, the pericardial cells were also injured in the FG1 and FG2 groups compared to the control groups. These cells have a mesodermal origin and are responsible for capturing, filtering, and eliminating toxic components in the hemolymph [48,54]. According to Mills and King [36], and confirmed in bees by other studies [37,44,51,55,56], the pericardial cells have four stages of activation related to stressful conditions. The presence of altered pericardial cells (stages II, III, and IV) identified in the FG1 and FG2 groups, with high cytoplasmic vacuolization and resulting peripheral displacement of the nucleus, suggests a great activity in the uptake of substances from the hemolymph [37]. They are corroborated as described by Balsamo et al. [44], who reported the same effects caused by thiamethoxam in young individuals of the species.

Additionally, it is important to highlight that the outcomes obtained in this present work come from exposure to the active principle alone—pyraclostrobin. However, commercial fungicide formulation may also impact the bees and the ecosystem “in situ”. Tadei et al. [32] highlighted that exposure to the pyraclostrobin active ingredient and Comet^®^ (commercial formulation-pyraclostrobin 250 g/L) in Africanized honey bee larvae induced midgut cytotoxicity, confirmed by immunohistochemical techniques (DNA fragmentation and HSP70). In the same way, Carneiro et al. [28] showed side effects on the midgut of nontarget organisms (*A. mellifera*) after exposure to Rovral^®^ SC (iprodione 500 g/L active ingredient).

Overall, studies of the sublethal effects of pesticides on bees still represent a low amount, and even so, most of the research focuses only on insecticides, leaving a worrying gap of 84% of fungicides with no data on sublethal effects (lowest observable adverse effect level (LOAEL)) [57]. From this perspective, the results of the current study are highly relevant for determining the possible environmental impacts of fungicide in a high-accuracy biomarker for the ecology of stress.

## 5. Conclusions

The data presented here highlighted the harmful effects of fungicide pyraclostrobin, even in residual concentrations, on individual foragers’ fitness, with potentially harmful effects at the colony level. The used biomarkers show high sensitivity at the cellular level and are suitable parameters to be used in future toxicological studies. In conclusion, the present study revealed that the fungicide potentially affected cells involved in the bee’s intermediary metabolism (fat body) and excretion of toxicants (pericardial cells), which contributed to reducing knowledge gaps about the sublethal effects of fungicides on bees.

## Data Availability

Raw data and all metadata associated with this research publication are available from Caio EC Domingues (caio.da@um.si).
